# Genome-Wide Alteration of 5-Hydroxymethylcytosine in Hypoxic-Ischemic Neonatal Rat Model of Cerebral Palsy

**DOI:** 10.3389/fnmol.2019.00214

**Published:** 2019-09-04

**Authors:** Yunpeng Zhang, Yaodong Zhang, Danmei Chen, Cuiting Wang, Long Chen, Chao Gao, Wei Fan, Jimin Shi, Jihong Zhang, Bing Li

**Affiliations:** ^1^Research Center for Clinical Medicine, Jinshan Hospital Affiliated to Fudan University, Shanghai, China; ^2^Department of Pediatrics, Children’s Hospital Affiliated to Zhengzhou University, Henan Children’s Hospital, Zhengzhou Children’s Hospital, Zhengzhou, China; ^3^Department of Neurology, Jinshan Hospital Affiliated to Fudan University, Shanghai, China; ^4^Department of Rehabilitation, Children’s Hospital Affiliated to Zhengzhou University, Henan Children’s Hospital, Zhengzhou Children’s Hospital, Zhengzhou, China

**Keywords:** cerebral palsy, hypoxic-ischemic injury, 5-hydroxymethylcytosine, DNA hydroxymethylation, Tet, neurodevelopment

## Abstract

Cerebral palsy (CP) is a neurodevelopmental disorder usually occurring early in life and persisting through the whole life. Several risk factors, including perinatal hypoxia-ischemia (HI), may contribute to occurrence of CP in preterm infants. DNA hydroxymethylation has been shown to play an important role in neurodevelopment and neurodegenerative disorders. However, the effect of DNA hydroxymethylation in CP remains unknown. The aim of this study is to explore whether and how DNA hydroxymethylation is involved in CP pathogenesis. We observed that overall 5-hydroxymethylcytosine (5hmC) abundance in the cortex of the temporal lobe of rat pups was decreased significantly after hypoxic-ischemic injury, and the reduced expression of Tet1 and Tet2 enzymes might be responsible for this change. Identified differential hydroxymethylation regions (DhMRs) were richly involved in multiple signaling pathways related to neuronal development and function. Furthermore, we found that reduced 5hmC modification on the DhMRs-related genes were accompanied by decrease of their mRNA expression levels. These results suggest that 5hmC modifications are involved in the CP pathogenesis and may potentially serve as a new therapeutic target.

## Introduction

Cerebral palsy is the most common cause for childhood mortality which shows a worldwide prevalence being around 2 to 3.5 death per 1000 live births. CP is characterized by motor and postural impairments and associated with cognitive and learning deficits (Colver et al., 2014). Perinatal cerebral HI injury increasing the risk of developmental malformations of CNS in the newborn is a major cause of CP (Kurinczuk et al., 2010). To date, a lot of basic science studies has utilized HI neonatal rat model to investigate the behavioral outcomes and pathogenesis of CP (Tai et al., 2009; Kim et al., 2018). However, the causative mechanisms of hypoxia-ischemia affecting development of CP still remain largely elusive.

Epigenetic modifications result in stable and inheritable changes in gene expression patterns without alteration in the coding sequences, which has profound impacts on the development of diseases (Murrell et al., 2013). Among all the known epigenetic mechanisms, DNA methylation is one of the most extensively studied and plays a critical role in chromatin structure remolding, transcriptional repression of genes, and embryonic development (Li et al., 1992; Zemach et al., 2010; Jursch et al., 2013). Furtherly, DNA methylation on the fifth carbon of cytosine (5mC) is essential for neurogenesis (Wang et al., 2016), learning and memory (Yu et al., 2011), and synaptic plasticity (Munoz et al., 2016) in mammalian CNS.

DNA methylation was long viewed as a permanent modification since it was established in the embryonic stage until the discovery of 5hmC in Kriaucionis and Heintz (2009) and Tahiliani et al. (2009). It is indicated by several studies that 5hmC is particularly rich in the brain than in other tissues of the body (Song et al., 2011; Khare et al., 2012), which suggests the possible functional importance of 5hmC in the brain. 5hmC is an oxidation product of 5mC which is catalyzed by the TET family of proteins (Ito et al., 2010). 5hmC not only acts as an intermediate during the removal of 5mC by passive or active demethylation processes (16), but also serves as a stable epigenetic mark during development of diseases (Tan and Shi, 2012). Alternation in the 5hmC profile of genomic DNA has been recently linked to some neurological disorders such as Alzheimer’s disease (Shu et al., 2016), Huntington’s disease (Wang et al., 2013), fragile X-associated tremor/ataxia syndrome (Yao et al., 2014), and the autism spectrum disorders (Papale et al., 2015). All these findings suggest the important role of 5hmC modification in neurodevelopmental and neurodegenerative disorders.

Despite the obvious occurrence of DNA hydroxymethylation in neurodevelopment, whether and how 5hmC is involved in CP pathogenesis is still mostly unknown. Thus, we applied an established chemical labeling and affinity purification method coupled with high-throughput sequencing technology to explore the genome-wide profiles of 5hmC in hypoxic-ischemically injured rat brain and its association with CP. We provided a genome-wide map of 5hmC profiles in a HI rat model of CP which serves to reveal known and potentially novel genes contributing to CP phenotype. These profiles provide new insight into the possible fact that DNA hydroxymethylation may contribute to CP pathogenesis, and 5hmC may be used as a potential biomarker and a therapeutic target for the treatment of CP.

## Materials and Methods

### Animals

Pregnant Sprague-Dawley rats weighing 340–380 g were purchased from the Slac Laboratory Animal Company (Shanghai, China). These Rats were free to give birth and the male pups were used for the studies. Animal care and treatment was performed in accordance with the Guidelines for Animal Experiments of the Chinese Academy of Medical Sciences, and with the approval from the Ethics Committee for Animal Care at Jinshan Hospital of Fudan University. All the rats were raised at a temperature (20–22°C) and humidity (55–65%) controlled environment with a constant 12-h light/dark cycle and allowed free access to adequate food and water. We measured the body weight of the rat pups from P4 to P28 (day of birth = P1).

### Hypoxia-Ischemia (HI) and Sham Treatments

Male rat pups were randomly divided into sham group and HI group. On postnatal day 3 (P3), rat pups underwent hypoxic-ischemic brain injury or sham treatments using the Levine model (Levine, 1960), as described by Rice et al. (1981). HI injury was induced by a permanent unilateral (left) CCA ligation with suture under isoflurane anesthesia. Total time of surgery never exceeded 10 min. Sham animals suffered the same procedure except for the ligation of the artery. After recovery for 1 h, the HI group were placed in a hypoxic chamber (92% N_2_, 8% O_2_) at 37°C for 3.5 h. Sham animals were treated identically, except that they were exposed to normal air (normoxic). Hypoxia-Ischemia (HI) and Sham Treatments. The number of the rats involved in different biochemical detection was shown in Supplementary Figure S1.

### Righting Reflex Test

Righting reflex was measured from the day of P5 to the day of P11 for each rat. Rat pups were placed in the supine position, and the time that it took for rat pups to turn over to the prone position with all the four paws in contact with the ground was recorded (Comhair et al., 2018).

### Step-Down Test

Step-down test was performed to measure memory on the rat pups, the process of which is as follows: a rat was placed on a cylindrical insulation platform (4.5 cm in diameter, 4.5 cm in height) in a test box (20 cm × 20 cm × 60 cm) to adapt to the surroundings for 3 min. A copper grid was placed on the bottom of box, and 0.8 mA of alternating current was continuously delivered to the device. The rat pups first received training session on P26 and test session on P27. In the 5-min -training session (P26), after stepping down, the animals received a foot shock and jumped back onto the platform immediately. In the test session (24 h after training, i.e., P27), the latency time (stepping down from the platform to the electric grid for the first time) and the number of errors (frequency of stepping down from the platform to the electric grid) were recorded.

### Morris Water Maze (MWM) Test

The MWM test was performed on the rat pups of P27 (1 day after step-down test). This test is widely used to assess spatial learning and memory (Morris, 1984) in animal models. The water maze was filled with 20–22°C water at controlled room temperature (22–26°C). The maze was divided into four quadrants, and each quadrant was marked with a different symbol to navigate. A transparent platform (5 cm diameter) was centered in one quadrant and fixed 1 cm below the water level. In training sessions, each rat was subjected to training four times a day for four consecutive days from P27 to P30. On the probe trial day (P31), the platform was removed and the rats were placed into the pool from the same entry point. The motion tracks and the number of platform location crossings were recorded and analyzed by animal behavior analysis system (Shanghai Xinruan Information Technology Company, Shanghai, China).

### Dot Blotting

All rat pups were sacrificed on P31 and the brains were removed. Genomic DNA was extracted from the left cerebral temporal lobe cortex by a AllPrep DNA Mini Kit (Qiangen, Germany). The procedure of DNA dot blot was conducted as previously described (Ning et al., 2016). The genomic DNA were denatured at 95°C and instantly placed on the ice for 5 min. 2.5 μL genomic DNA (100 ng/uL) were added on positively charged nylon membranes. After being baked at 80°C for 30 min to cross-link DNA, the membrane was blocked with 5% skimmed milk. Next, the membrane was incubated with 5-hmC primary antibody (1:1000 dilution, Active Motif, United States, Cat. No. 39769) at 4°C overnight. Then, the membrane was treated with the HRP-conjugated secondary antibody, labeled with ECL reagent and stained with 0.02% methylene blue to ensure equal loading.

### Hydroxymethylated DNA Immunoprecipitation (hMeDIP) Analysis and High-Throughput Sequencing

Genomic DNA was isolated from the left temporal cortex of six rat pups (control: *n* = 3; HI: *n* = 3) and sonicated to 100–200 base pairs (bp) which was sequenced using the Illumina Hiseq2000 and Nextseq system (Illumina, United States) in accordance with the manufacturer’s instructions. To prepare for hMeDIP followed by next-generation sequencing (hMeDIP-seq), DNA fragments were first ligated with Illumina adaptors for further amplification. Subsequently, the denaturation of dsDNA was performed at 95°C for 10 min and immunoprecipitated by using an anti-5hmC antibody (Active Motif, United States, Cat. No. 39769). Lastly, the enriched hydroxymethylated DNA fragments were amplified by polymerase chain reaction (PCR) and high-throughput sequencing was subsequently performed on the Illumina HiSeq2000.

### Sequence Alignment and Mapped Reads Annotation

Bioinformatics data was analyzed as described in previous papers (Kim et al., 2016). Briefly, FASTQ sequence files obtained were aligned to the reference genome of rats in UCSC databank (RGSC6.0/rn6) using Bowtie (Langmead et al., 2009). Only unique non-duplicate reads were used for peak calling and annotation by HOMER (hypergeometric optimization of motif enrichment) software (Heinz et al., 2010). DhMRs between sham and HI rats were determined by comparing 5hmC peak levels by MACS software (Zhang et al., 2008). DhMRs were identified by directly comparing one to the other, rather than comparing to the input (FDR cutoff = 0.05). GO analysis was performed with DAVID (Huang da et al., 2009).

### Real-Time PCR

Total RNA was extracted from the left cerebral temporal lobe cortex of rat pups. Reverse transcription of cDNA was performed using the PrimeScript RT Master Mix Kit (Takara, China) according to the manufacturer’s instructions. And Real-time PCR analysis was performed with SYBR green (Takara, China) on the 7500 Real-Time PCR system (Applied Biosystems, United States). β-Actin was used as an endogenous control and relative expression of target gene was determined by the 2^–ΔΔCT^ method. The specific primers were listed as follows: Zrsr2 primer (forward 5′-GTCCTGCCTGAGTTCAAGAGTGTG-3′, reverse 5′-GAAGCTGTCGTCCTGCATACCATC-3′), Slc44a1 primer (forward 5′-ACACAGCCACAGCCATCAATAGC-3′, reverse 5′-CAGCCACTCGCAGAGCATTCTC-3′), Casd1 primer (forward 5′-GAGAGCAGACGGATGAATGGAAGG-3′, reverse 5′-AACAGATAAGCAGCCACCAGAACG-3′) and β-actin primer (forward 5′-CATGTACGTTGCTATCCAGGC-3′, reverse 5′-CTCCTTAATGTCACGCACGAT-3′).

### Western Blot Assay

For western blot analyses, the left temporal lobe cortex tissues were first homogenized with ice-cold lysis buffer and then centrifuged at 12,000 rpm for 20 min at 4°C. Lastly, the supernatant was collected for protein content analysis. An equal amount of protein (20 ug) for each sample was loaded onto an 8 or 10% sodium dodecyl sulfate (SDS)–polyacrylamide gel and separated by electrophoresis. Then, proteins were transferred onto a polyvinylidene difluoride (PVDF) membrane (Millipore, United States). After being blocked with 5% skimmed milk for 2 h, the membrane was incubated with different primary antibodies in TBST overnight at 4°C, including rat anti-TET1 (1:1000, Abcam, United Kingdom, Cat. No. 191698), rabbit anti-TET2 (1:1000, Millipore, United States, Cat. No. ABE364) and rabbit anti-β tubulin (1:2000, Cell Signaling Technology, United States, Cat. No. 2128). After three times wash with TBST, the membrane was incubated with HRP-conjugated secondary antibody for 1 h at room temperature and probed using ECL reagent.

### Immunofluorescence Confocal Microscopy

A total of 10 rat pups (Sham: *n* = 5; HI: *n* = 5) were sacrificed on P31 followed by immediate intracardial perfusion with 4% paraformaldehyde (PFA). The brains were removed and embedded into paraffin. Serial sections (5 μm thick) were cut through the cerebral temporal lobe cortex. After microwave antigen retrieval in citric acid buffer at 95°C, these brain sections were then blocked with bovine serum albumin (BSA) for 1h at room temperature and incubated with 5hmC primary rabbit antibody (1:1000, Active Motif, United States, Cat. No. 39769) at 4°C overnight. Subsequently, these sections were then washed by PBS buffer for three times, which was followed by incubation in secondary Alexa Fluor 594 donkey anti-rabbit IgG (1:200, Life Technology, United States) antibody for 1h at 37°C. And 4′, 6-diamino-2-phenylindole (DAPI) were used for nuclear immunofluorescence staining. Lastly, the produced immunofluorescence was visualized and captured on a confocal microscope (Leica sp5, Germany).

### Statistical Analysis

**FIGURE 1:**
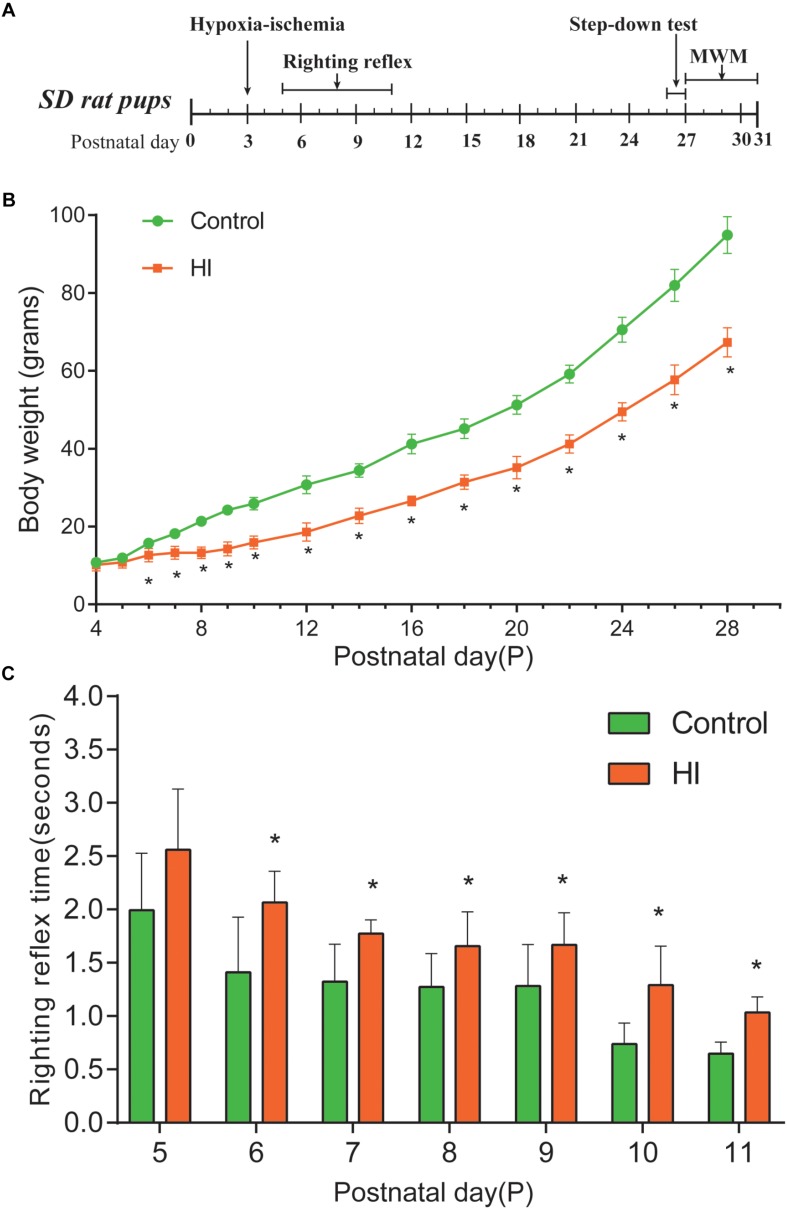
Hypoxia-ischemia induced growth retardation and neurodevelopment deficits. **(A)** Experimental timeline. **(B)** Average body weight of control and hypoxic–ischemic pups measured from day 4 (P4) until the day 28 (i.e., 1 day after the hypoxic-ischemic interference, *n* = 8). **(C)** Mean righting reflex time of HI and control rats (*n* = 8). ^∗^*P* < 0.05, versus control group.

Quantitative data are expressed as the means ± standard deviations (SD). All the data were analyzed by using SPSS 15.0. The independent sample *t*-test and two-way analysis of variance (ANOVA) was applied to determine the differences in body weight and righting reflex time in Figure 1; a Mann–Whitney *U*-test (also known as a Wilcoxon rank-sum test) was performed on each gene region to test whether the median 5hmC level of HI group differed from control group in Figure 4; other results were determined by independent sample *t*-test. *P-*values < 0.05 was considered statistically significant.

**FIGURE 2:**
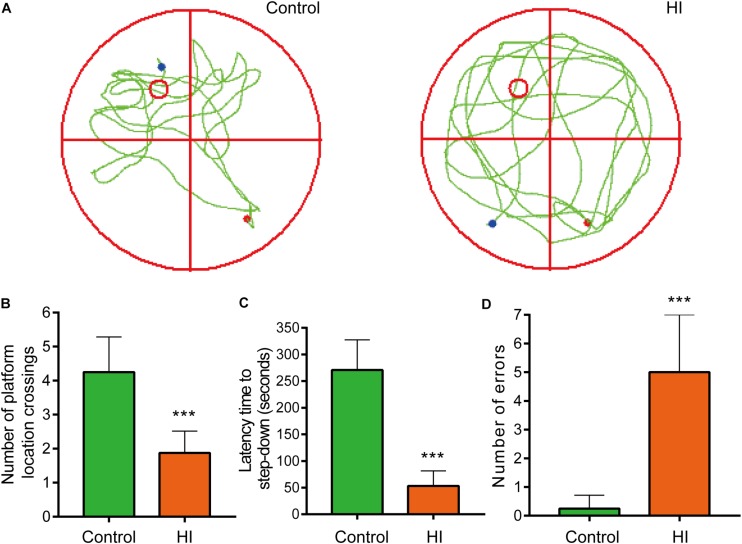
Hypoxia-ischemia impaired learning and memory functions of rat pups (*n* = 8). **(A)** Motion trails of two groups. **(B)** The average number of platform location crossings during the probe trial of MWM test. **(C)** The latency time of step-down test. **(D)** The errors number of step-down test. ^∗∗∗^*P* < 0.001, versus control group.

**FIGURE 3:**
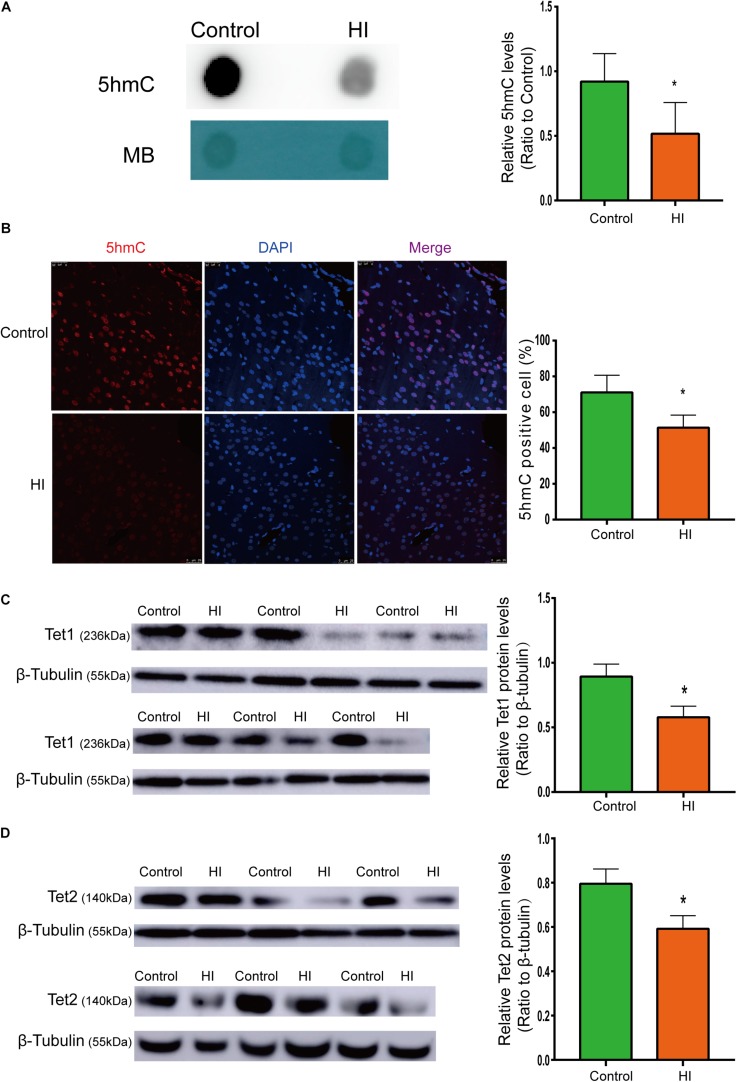
Reduced 5hmC and Tet proteins level in HI rat cortex. **(A)** Representative dot blotting results of genomic 5hmC (*n* = 5). Methylene blue staining was used as a loading control. **(B)** Immunofluorescence staining with 5hmC antibody in the left temporal cortex (*n* = 5). 5hmC was labeled with Alexa Fluor 594 (red), and the neuron nuclei were labeled with DAPI (blue). The expression level of Tet1 **(C)** andTet2 **(D)** protein by western blotting (*n* = 6). ^∗^*P* < 0.05 versus control group.

**FIGURE 4:**
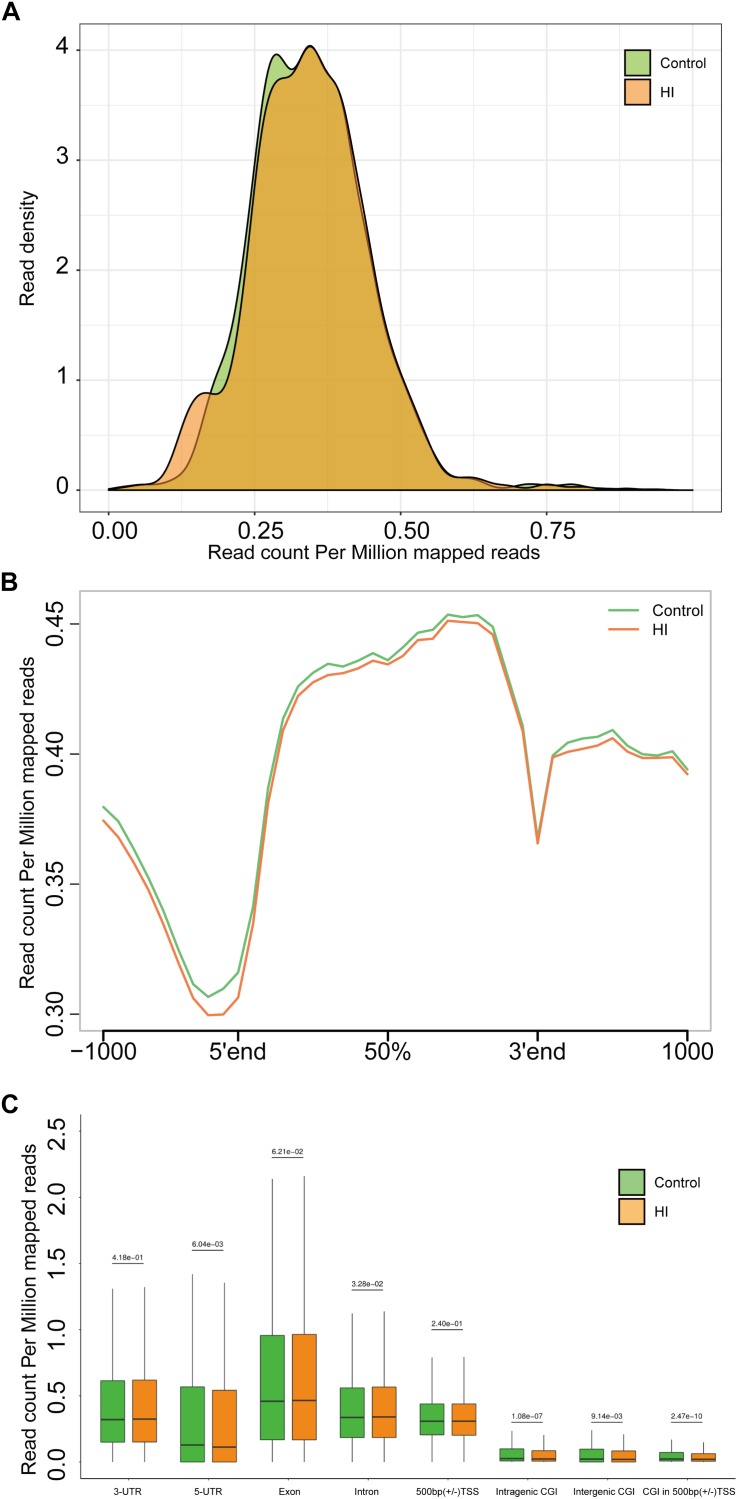
**(A)** Genome-wide 5hmC reads density distribution. **(B)** Normalized 5hmC reads densities across TSS, TES, and RefSeq gene bodies. Gene bodies were normalized to 0–100% as relative positions. **(C)** Normalized 5hmC densities overlapping with known genomic features. Above the horizontal line is the *P*-value compared between the control group and the HI group.

### Accession Number

Sequencing data have been deposited to the GEO in NCBI with Accession No. GSE133787.

## Results

### HI Rat Pups Displayed Weight Loss and Righting Reflex Disorder

The body weight of rat pups was measured from the day of P4 to P28. Figure 1A is our experimental timeline. As shown in Figure 1B, from P6 to P28, the body weight of the rats in the HI group decreased significantly compared to that of the control group (*n* = 8; *p* < 0.05). Then, we assessed the impact of neonatal HI brain injury on the righting reflex to determine whether there were any deficits in this fundamental nervous reflex. As a result, the righting reflex time of HI rats was significantly longer than that of control rats from P6 to P11 (*n* = 8; *p* < 0.05; Figure 1C).

### Hypoxia-Ischemia Resulted in Significant Learning and Memory Disorders of the Rat Pups

To further evaluate the learning and memory functions of the rats, we conducted MWM test and step-down test. In the probe trial of MWM test, motion tracks of the rats were shown in Figure 2A. Consequently, the number of platform location crossings of HI group was less than that of control group, indicating the impairment of spatial memory capacity (*n* = 8; *p* < 0.001; Figure 2B). In step-down test, the latency time was used to assess learning and memory ability of the rats. The longer one indicates greater preference for learning and memory. As a result, the HI rats showed significantly shorter latency time and more errors compared to control rats (*n* = 8; *p* < 0.001; Figures 2C,D).

### Hypoxic-Ischemic Injury Decreased 5hmc Level in Rat Cerebral Temporal Cortex

To examine whether the level of 5hmC is affected during CP pathogenesis, we examined 5hmC levels in the left temporal cortex dissected from HI model and control rats on P31 by dot blotting. As shown in Figure 3A, the 5hmC level of HI rats decreased significantly compared to that of control rats (*n* = 5; *p* < 0.05). In Figure 3B, 5hmC immunofluorescence staining overlaps with intranuclear DAPI staining. Compared with control rats, HI rats revealed lower 5hmC level in the left temporal cortex (*n* = 5; *p* < 0.05). Hence, immunofluorescence displayed similar trend as dot blotting result.

### Tet1 and Tet2 Expression Was Down-Regulated in Hypoxic-Ischemic Rat Brain

Because 5hmC is produced via oxidation of 5mC by proteins of Tet family, we then investigated protein expression levels of Tet1 and Tet2. As shown by western blotting results (Figures 3C,D), the expression of Tet1 and Tet2 was significantly decreased after HI injury (n = 6; p < 0.05).

### Hypoxic-Ischemic Injury Caused Genome-Wide 5hmc Alteration

The results above suggested a global decrease in 5hmc level in HI rats compared to that in control rats. To furtherly determine the exact location and distribution of genome-wide 5hmc, we employed an established chemical conjugation and affinity purification method coupled with high-throughput sequencing technology (Szulwach et al., 2011). Three hypoxic-ischemic rats and three matched control rats were sacrificed on P31 and genomic DNA was extracted from the left cerebral temporal lobe cortex for analyses. In Figure 4A, genome-wide pattern of 5hmC levels were evaluated by counting 5hmC-mapped reads in each 100 kb bin from control and HI samples and then normalized to the total sequencing coverage. Furthermore, the distribution of 5hmC reads was studied at 1kb up- and down-stream of gene bodies (Figure 4B). From Figures 4A,B, we found that the distribution of the genome-wide 5hmC reads in control and HI groups was significantly different, and we then explored the specific gene regions in which this difference existed. Figure 4C showed the overlapping features of normalized densities of 5hmC reads in control and HI groups with known genomic features on the defined gene bodies and CGI (CpG islands) obtained from the UCSC Tables for RGSC6.0/rn6. It was found that 5hmC mapped reads were significantly reduced on intragenic CGI, intergenic CGI, and CGI in 500bp (±) of transcriptional start sites (TSS) after HI injury.

### Identification and Characterization of Differentially Hydroxymethylated Regions (DhMRs) After HI Injury

**FIGURE 5:**
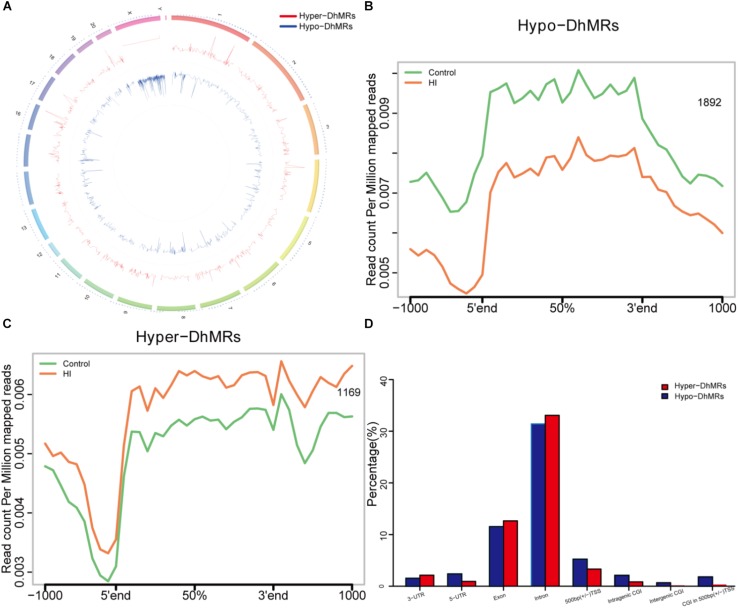
Identification and characterization of differentially hydroxymethylated regions (DhMRs) in hypoxic-ischemic rat models. **(A)** Chromosome circular map of genome-wide DhMRs. **(B)** Identification of 1892 hypo-DhMRs. **(C)** Identification of 1169 hyper-DhMRs. **(D)** Annotation of DhMRs to various genomic features.

Since our present data suggested distinct 5hmC distribution pattern, we proceeded to identify and characterize the control and HI specific DhMRs across the genome, aiming to recognize specific loci that exhibited altered 5hmC profiles between the control and HI rats. Consequently, it was found a total of 3061 DhMRs using diffReps (Shen et al., 2013), with 1169 HI-specifically increased hydroxymethylation (hyper-DhMRs) and 1892 HI-specifically decreased hydroxymethylation (hypo-DhMRs) in the HI model as compared to those of control group, were distributed across the genome (Figures 5A–C). The lower number of increased HI-specific hyper-DhMRs was consistent with the global reduction of 5hmC in HI rats presented in Figure 4A. Remarkably, hyper-DhMRs showed a notable decrease on X chromosome compared to hypo-DhMRs in the chromosome circular map (Figure 5A).

To explore the known genomic features associated with the identified DhMRs, we annotated all the DhMRs of the rat genome using HOMER (Hypergeometric Optimization of Motif Enrichment) software. HOMER annotations revealed that the identified hypo- and hyper- DhMRs displayed the similar distribution trend: abundantly rich in exon and intron (Figure 5D), suggesting a high conservation during CP progress. Whereas, the distributions of increased HI-specific hyper-DhMRs on intragenic CGI, intergenic CGI, CGI within 500 bp of TSS and ±500 bp of TSS showed the same depletion pattern as the distributions of 5hmC reads in Figure 4B after HI injury.

### Annotation of DhMRs Revealed Known and Potentially Novel Cerebral Palsy Genes

**FIGURE 6:**
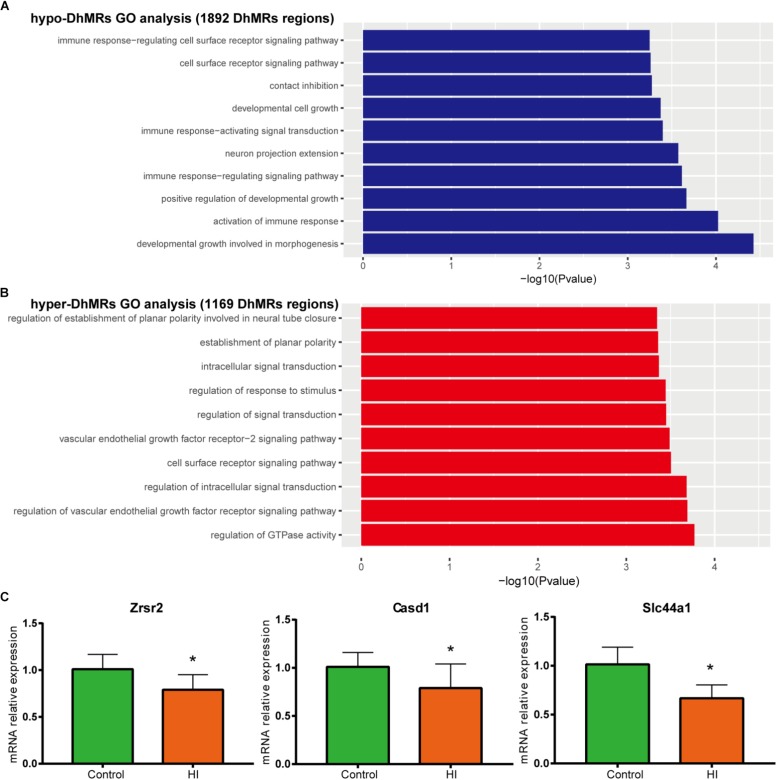
Gene oncology analyses for DhMRs. **(A)** Top 10 GO biological processes associated with hypo-DhMRs-associated genes. **(B)** Top 10 GO BPs associated with hyper-DhMRs-associated genes. **(C)** RT-PCR analysis of the mRNA levels of selected hypo-DhMRs related genes in control and HI rats. β-Actin was used as a control. ^∗^*P* < 0.05, versus control group.

To further examine the biological significance of the association of the found DhMRs with those identified genes, we performed GO analyses of the hyper- and hypo- DhMRs, respectively. Remarkably, several GO biological processes (BPs) associated with neuronal function and development of the brain were found (Figures 6A,B) which included regulation of cell communication in hyper-DhMRs, as well as cell developmental growth, neuron projections extension, positive regulation of developmental growth and developmental growth involved in morphogenesis of hypo-DhMRs.

Annotation of all the DhMRs to genes revealed altogether 951 genes and we compared them with a recently list of known CP-related genes (*N* = 1679, obtained from GeneCards database). As a result, it was found that a significant number of DhMRs-related genes were overlapping with some known CP genes (*N* = 88 of 951), including Notch1, Slc16a2, Dmd, Grin2b. Previous studies also revealed that the increased 5hmC modification in genes coincides with cell-specific active gene transcription (Mellen et al., 2012). To explore this further, we examined the expression levels of some hypo-DhMRs- related genes associated to the nervous system. Accompanied by reduced 5hmC modification in genes, the mRNA levels of Casd1, Zrsr2, and Slc44a1 were significantly decreased after HI injury (Figure 6C). Taken this together, the dynamic change of 5hmC modification may contribute to the CP pathogenesis by regulating related genes’ transcription.

## Discussion

Environmentally sensitive epigenetic modification is emerging as a significant mechanism in the molecular pathogenesis of many diseases. There are various ways of epigenetic modification, such as methylation, demethylation, and acetylation, etc. As the product of a new epigenetic modification, hydroxymethylation, 5hmC is about 10-fold more abundant in the neurons than in all the other cells, which is indicative of its significant role in postnatal neurodevelopment (Szulwach et al., 2011; Khare et al., 2012; Wang et al., 2012). In addition, more evidence demonstrates that modification of 5hmC contributes to many neurological disorders, including Huntington’s disease, Alzheimer’s disease, the autism spectrum disorders, and fragile X-associated tremor/ataxia syndrome (Wang et al., 2013; Cadena-del-Castillo et al., 2014; Yao et al., 2014; Papale et al., 2015; Shu et al., 2016). Despite the recent growing interest in 5hmC modifications, whether 5hmC is involved in CP is still unexplored.

In our study, we found that overall 5hmC abundance was significantly decreased in the hypoxic-ischemic brain injury model, which was consistent with the reduced 5hmC level after ischemia reperfusion in the mouse kidney in the previous report (Huang et al., 2012). And we also observed a decrease in Tet1 and Tet2 enzyme expression simultaneously, which might be responsible for the decrease of 5hmC. On the other hand, it was found that ischemia-hypoxia often occurs in the malignant tumors when angiogenesis is not sufficient to maintain their growth (Hanahan and Weinberg, 2011). Low levels of 5hmC and Tet proteins were confirmed in many studies on this aspect (Jin et al., 2011; Yang et al., 2013). Due to the similar hypoxic-ischemic pathophysiology mechanism, hypoxic-ischemic-induced brain injury and ischemia-hypoxia-related tumor growth retardation are likely to share some similar regulation mechanisms in the down-regulation of 5hmC level.

Previous studies have found 5hmC can be very dynamic during postnatal neurodevelopment (Szulwach et al., 2011; Lister et al., 2013). The data presented here are consistent with those findings. Our genome-wide 5hmC distribution profiling studies suggested that the enrichment of 5hmC in intragenic CGI, intergenic CGI, and CGI in 500 bp **(±)** of TSS was decreased after HI injury. It was also observed that CGI methylation is critical in gene silencing, and 5hmC, as a marker of active genes, may play a role in gene expression mediated by DNA demethylation (Deaton and Bird, 2011; Li et al., 2016). In line with these findings, our RT-PCR results showed that the depletion of 5hmC modification in Casd1, Zrsr2, and Slc44a1 gene were accompanied by reduced mRNA levels of these genes. All these findings demonstrate a possible novel pathogenesis mechanism of CP, in which 5hmC modification may play a critical role in regulating the expression of neuron-function-related genes and possibly be implicated in CP pathogenesis.

Using hMeDIP sequencing techniques, we found 951 hydroxymethylation differential genes. In addition to the proven genes whose expression levels are accompanied by a decrease in the level of hydroxymethylation, we also found that 88 differential genes are known to be involved in the pathogenesis of CP (compared to the GeneCards database). For example, Notch1, Slc16a2, Dmd, Grin2b, Slc1a1, Bmp6, Aff2, Crb1 and Phex, the hydroxylation status of these genes known to be involved in the onset of CP has been significantly different after HI injury. Previous studies found that changes in the level of 5hmc are closely related to the occurrence of many neurological diseases (Wang et al., 2012; Miao et al., 2015; Zhao et al., 2017). Perhaps the hydroxymethylation changes of these genes may be related to their relationship with CP.

In the cohorts of children suffering from CP and related neurodevelopmental disorders, it was found that male kids typically outnumber female kids whereas the reasons for this disparity are uncertain (Johnston and Hagberg, 2007; Reid et al., 2016). In the neonatal mouse model of HI brain injury, the same gender-specific cerebral protective effect was observed in the female mice (Pimentel-Coelho et al., 2013). Based on these research results, only male rat pups were selected for our current study. Notably, it was also shown in our results that X chromosome distribution of HI-specifically increased hyper-DhMRs decreased dramatically compared with HI-specifically decreased hypo-DhMRs (Figure 5A) which is consistent with the previous studies to furtherly confirm the correlation between sex and occurrence of CP.

Furthermore, GO biological processes enrichment analyses identified many DhMRs-associated genes which are rich in multiple signaling pathways related to neurodevelopment and neuronal function, indicating the correlation between 5-hydroxymethylcytosine loci, neuronal development and ultimate brain functions. In our animal experiments, HI rat pups presented growth retardation and neurodevelopmental disorders. Thus, it is noteworthy that the relationship between hydroxymethylation and injured neurodevelopment in the case of CP may be present.

## Conclusion

Our results revealed that hypoxic-ischemic brain injury decreased the overall 5hmC abundance in rat temporal cortex, which may correlate with the reduced expression of Tet1 and Tet2. Meanwhile, genome-wide analyses also displayed large scale of 5hmC alterations among the genes involved in neurodevelopment and the depletion of 5hmC modifications in DhMRs-associated genes are accompanied by reduced mRNA level of these genes, which may altogether contribute to finding those potentially abnormally expressed genes associated with the occurrence of CP phenotype. In summary, the possible correlation between DNA hydroxymethylation and 5hmC dynamic change may shed light on possible novel pathogenesis mechanisms of CP. To understand how altered DNA demethylation affects the onset and development of CP will be the focus for our future research.

## Ethics Statement

This study was carried out in accordance with the Guidelines for Animal Experiments of the Chinese Academy of Medical Sciences, and with the approval from the Ethics Committee for Animal Care at Jinshan Hospital of Fudan University.

## Author Contributions

YuZ and YaZ supervised the experiments and analyzed the data. DC, CW, LC, CG, WF, JS, and JZ conducted the experiments and recorded the data. BL designed the study, drafted the manuscript, and analyzed the data.

## Conflict of Interest Statement

The authors declare that the research was conducted in the absence of any commercial or financial relationships that could be construed as a potential conflict of interest.

## Supplementary Material

FIGURE S1 The number of animals used in the specific experiment. In Control and HIgroups, behavioral tests (righting reflex, Morris water maze test, and step-down test)were performed on a total of 8 rats in each group. Then, 6 of the 8 rats were used forDB and WB analysis (the remaining two were included in other experiments).Subsequently, half of the temporal cortex of the 6 rats were used to extract protein forWB and the other half to extract genomic DNA. Genomic DNA was used to examine5hmC levels by dot blotting, and three samples with sufficient DNA were subjected tohMeDIP-seq. Compared with control group, the expression of Tet protein and 5hmCin the three mice used for sequencing significantly decreased in HI group. Meanwhile, the brain tissue of five rats was sliced for immunofluorescence detection.

## Abbreviations

CNS: central nervous system
CP: cerebral palsy
CpG: cytosine guanine
DhMRs: differential hydroxymethylation regions
FDR: false discovery rate
GO: gene ontology
HI: hypoxia-ischemia
hMeDIP: hydroxymethylated DNA immunoprecipitation
hMeDIP-seq: hydroxymethylated DNA immunoprecipitation sequencing
HOMER: hypergeometric optimization of motif enrichment
5hmC: 5-hydroxymethylcytosine
5mC: 5-methylcytosine
MACS: model-based analysis of ChIP-Seq
MWM: Morris water maze
Tet: ten-eleven translocation.
