# Supplementary figures and images for: Genome-Wide Alteration of 5-Hydroxymethylcytosine in Hypoxic-Ischemic Neonatal Rat Model of Cerebral Palsy
Source: Front Mol Neurosci. 2019 Sep 4;12:214. doi: 10.3389/fnmol.2019.00214 (PMC6737274; doi:10.3389/fnmol.2019.00214)

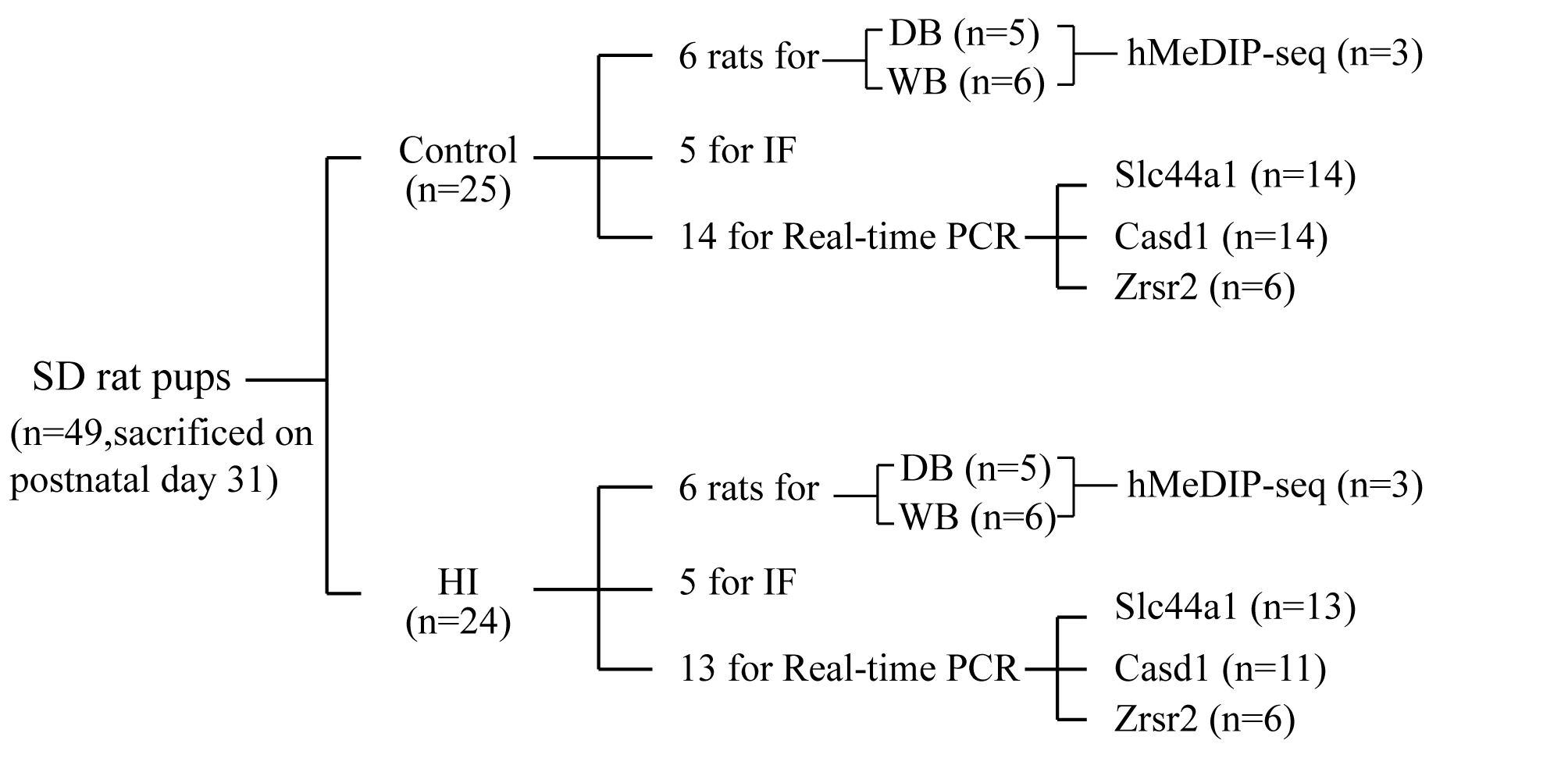

Supplement: FIGURE S1 — The number of animals used in the specific experiment. In Control and HIgroups, behavioral tests (righting reflex, Morris water maze test, and step-down test)were performed on a total of 8 rats in each group. Then, 6 of the 8 rats were used forDB and WB analysis (the remaining two were included in other experiments).Subsequently, half of the temporal cortex of the 6 rats were used to extract protein forWB and the other half to extract genomic DNA. Genomic DNA was used to examine5hmC levels by dot blotting, and three samples with sufficient DNA were subjected tohMeDIP-seq. Compared with control group, the expression of Tet protein and 5hmCin the three mice used for sequencing significantly decreased in HI group. Meanwhile, the brain tissue of five rats was sliced for immunofluorescence detection. [file Image_1.tif]
